# Mogroside V Protects Porcine Oocytes From Lipopolysaccharide-Induced Meiotic Defects

**DOI:** 10.3389/fcell.2021.639691

**Published:** 2021-03-02

**Authors:** Ke Yan, Kexin Cui, Junyu Nie, Hengye Zhang, Lumin Sui, Huiting Zhang, Xiaogan Yang, Chang-Long Xu, Xingwei Liang

**Affiliations:** ^1^State Key Laboratory for Conservation and Utilization of Subtropical Agro-bioresources, College of Animal Science and Technology, Guangxi University, Nanning, China; ^2^Reproductive Medical Center of Nanning Second People’s Hospital, Nanning, China

**Keywords:** oocyte, meiotic maturation, lipopolysaccharide, mogroside V, oxidative stress

## Abstract

Accumulating evidence has demonstrated that lipopolysaccharide (LPS) compromises female reproduction, especially oocyte maturation and competence. However, methods to protect oocyte quality from LPS-induced deterioration remain largely unexplored. We previously found that mogroside V (MV) can promote oocyte maturation and embryonic development. However, whether MV can alleviate the adverse effects of LPS exposure on oocyte maturation is unclear. Thus, in this study, we used porcine oocytes as a model to explore the effects of MV administration on LPS-induced oocyte meiotic defects. Our findings show that supplementation with MV protected oocytes from the LPS-mediated reduction in the meiotic maturation rate and the subsequent blastocyst formation rate. In addition, MV alleviated the abnormalities in spindle formation and chromosome alignment, decrease in α-tubulin acetylation levels, the disruption of actin polymerization, and the reductions in mitochondrial contents and lipid droplet contents caused by LPS exposure. Meanwhile, LPS reduced m^6^A levels in oocytes, but MV restored these epigenetic modifications. Furthermore, MV reduced reactive oxygen species (ROS) levels and early apoptosis in oocytes exposed to LPS. In summary, our study demonstrates that MV can protect oocytes from LPS-induced meiotic defects in part by reducing oxidative stress and maintaining m^6^A levels.

## Introduction

Lipopolysaccharide (LPS), the main component of the outer membrane of the cell wall in gram-negative bacteria, is a typical pathogen-associated molecular pattern (PAMP), also known as an endotoxin (Bidne et al., 2018). LPS is composed of lipid A, core polysaccharides, and O-antigens and is released via bacterial metabolism and after death/lysis. As a type of PAMP, LPS is recognized by immunocytes and promotes the release of cytokines, which can lead to systemic inflammation, multiple-organ dysfunction syndrome, endotoxemia, and septic shock (Cavaillon, 2018). LPS also adversely affects female reproduction; for example, it inhibits the secretion of reproductive hormones, disturbs the regulation of follicular activation, and reduces the primordial follicular pool reserve (Bromfield and Sheldon, 2013; Magata et al., 2014; Bidne et al., 2018). Collectively, it is shown that LPS severely affects physiological health and female reproductive performance.

High-quality oocytes are essential for proper embryonic development and successful reproductive outcome. Both *in vivo* and *in vitro* studies have shown that LPS treatment inhibits the meiotic maturation progress of oocytes (Magata and Shimizu, 2017; Shepel et al., 2018; Heidari et al., 2019) and further impairs embryonic developmental potential in diverse animal species (Magata and Shimizu, 2017; Zhao et al., 2017). In bovines and sheep, the addition of LPS to the *in vitro* maturation (IVM) medium induces oxidative stress, interferes with oocyte meiotic maturation (Zhao et al., 2017; Shepel et al., 2018), increases the apoptosis index of blastocysts, and affects early embryonic developmental competence (Roth et al., 2015; Magata and Shimizu, 2017). Oocyte quality and developmental competence are also decreased by LPS exposure in mice and rainbow trout (Crespo et al., 2010; Shepel et al., 2018). Although LPS toxicity toward oocytes and embryos has been reported in many species, the negative effects of LPS on mammalian oocyte maturation and embryonic developmental competence and the underlying mechanism still need to be further elucidated.

Mogroside V (MV), the most abundant mogroside in *Siraitia grosvenorii* (Luo-han-guo, LHG), has numerous pharmacological properties, such as antioxidative (Liu et al., 2018), antiobesity (Zhang et al., 2018), antidiabetic, anti-inflammatory, and anticancer properties (Weerawatanakorn et al., 2014). As a potent antioxidant, MV has significant oxygen free radical scavenging capacity and attenuates lipid peroxidation induced by Fe^2+^ or H_2_O_2_ (Li et al., 2014). In addition, our previous studies demonstrated that MV reduces reactive oxygen species (ROS) levels and promotes mitochondrial function to improve porcine oocyte IVM (Nie et al., 2020) and protects oocyte quality from deterioration during *in vitro* aging (Nie et al., 2019). Furthermore, protective effects of MV against LPS-induced diseases, such as alleviation of LPS-induced memory impairment and acute lung injury in mice and prevention of LPS-induced oxidative stress injury in RAW264.7 cells have been reported (Di et al., 2011; Shi et al., 2014; Li et al., 2019; Wang et al., 2020). In general, MV is a natural substance with many biological activities and has the potential to be an effective preventive agent against LPS-induced impairments.

However, whether MV can rescue oocytes from meiotic defects under LPS exposure remains unclear. In this study, we examined the effects of MV on porcine oocyte meiotic maturation, early embryonic development, cytoskeleton structure, mitochondrial contents, lipid droplet (LD) contents, oxidative stress level, and m^6^A modification in LPS-exposed oocytes. Our findings may provide a potential strategy for protecting oocytes from LPS-induced meiotic defects.

## Materials and Methods

The chemicals and reagents used in the present study were purchased from Sigma Chemical Co. (St. Louis, MO, United States) except when stated otherwise.

### Oocyte Collection and IVM

The animal experiments were approved by the Institutional Animal Care and Use Committee (IACUC) of Guangxi University and were conducted in accordance with the animal welfare and ethical rules. Ovaries were obtained from juvenile pigs at a local abattoir and transported to the laboratory in sterilized 0.9% saline solution at 32°C. After the ovaries were washed three times with prewarmed 0.9% saline solution, the cumulus–oocyte complexes (COCs) were aspirated from 3 to 8 mm antral follicles with a syringe fitted with an 18-gauge needle. Approximately forty COCs were cultured in a 200 μL modified bicarbonate-buffered TCM-199 medium, supplemented with 0.1% (w/v) polyvinyl alcohol (PVA), 3.05 mM d-glucose, 0.91 mM sodium pyruvate, 0.57 mM cysteine, 0.01 μg/mL epidermal growth factor, 0.50 μg/mL follicle-stimulating hormone, 0.50 μg/mL luteinizing hormone, 75.00 μg/mL penicillin G, 50.00 μg/mL streptomycin, and 10% porcine follicular fluid. The medium was covered with mineral oil and cultured for 26 or 44 h at 38.5°C in a 5% CO_2_ incubator with humidified air.

### LPS and MV Treatment

LPS was dissolved in sterilized water to a concentration of 100 mg/mL. MV (Chengdu Biopurify Phytochemicals, Ltd., Chengdu, China) was dissolved in dimethylsulfoxide (DMSO) to a concentration of 100 mM and diluted to a working concentration with IVM medium. The final concentration of DMSO in the IVM medium was less than 0.1%.

### Parthenogenetic Activation (PA) and *in vitro* Culture

To detect embryonic developmental potential, PA was performed using a BTX Elector-Cell Manipulator 2001 (BTX Inc., San Diego, CA, United States) according to the method described in a previous study (Nie et al., 2016). The activation medium contained 0.30 M mannitol, 1.00 mM CaCl_2_ ⋅ 2H_2_O, 0.10 mM MgSO_4_, 0.50 mM HEPES, and 0.3% (w/v) bovine serum albumin (BSA). After activation, the embryos were immediately transferred to PZM-3 medium (Yoshioka et al., 2002) and cultured at 38.5°C in a 5% CO_2_ incubator with humidified air. The cleavage rate (percentage of activated oocytes) and blastocyst formation rate (percentage of activated oocytes) were examined after 48 and 144 h, respectively.

### Immunofluorescence Staining and Confocal Microscopy

Cumulus cells were removed from COCs in polyvinyl alcohol (PVA)-TL-HEPES containing 0.1% hyaluronidase. The denuded oocytes (DOs) were washed three times in 0.1% PVA-Dulbecco’s phosphate-buffered saline (DPBS) and then fixed in 4% paraformaldehyde (PFA) for 30 min at room temperature (RT). The oocytes were permeabilized with 1% Triton X-100 in DPBS for 8–12 h at RT. Then, the oocytes were blocked with 1% BSA in DPBS for 1 h at RT and incubated with anti-α-tubulin FITC (1:150, Abcam, Cambridge, United Kingdom), anti-acetylated tubulin (1:100), phalloidin-FITC (1:400, AAT Bioquest, CA, United States), and rabbit monoclonal anti-methylation at the N6 position of adenosine (m^6^A) antibodies (1:400, Abcam) at 4°C overnight. After three washes with DPBS containing 0.1% Tween-20 and 0.01% Triton X-100, the oocytes were incubated with appropriate secondary antibodies for 1 h at RT. Then, the oocytes were washed three times and counterstained with 10 μg/mL propidium iodide (PI) or Hoechst 33342 for 10 min at RT. Finally, the oocytes were washed, immediately mounted on glass slides, and imaged under a confocal laser scanning microscope (TCS-SP8 with LAS, Leica, Wetzlar, Germany).

Spindle formation and chromosome alignment determination were double-blinded. For fluorescence intensity measurement, the same immunostaining procedure and the same scanning setting were used to acquire signals. Then, an identical size region of interest (ROI) in the cell membrane or cytoplasm was defined and fluorescence signals were calculated with ImageJ software (National Institutes of Health, Bethesda, MD, United States). Finally, the average values of all measurements of an individual group were used to compare fluorescence intensities.

### Determination of Mitochondrial Contents

Mitochondrial content was detected with an orange fluorescent dye (MitoTracker Orange CMTMRos, Thermo Fisher Scientific, MA, United States). After three washes in 0.1% PVA-DPBS, DOs were incubated with 200 nM orange-fluorescent dye for 30 min at 38.5°C and subsequently fixed in 4% PFA for 30 min at RT. After three washes with 0.1% PVA-DPBS, the oocytes were mounted on glass slides and imaged under a confocal microscope with the same scanning setting. The fluorescence intensity was calculated with ImageJ software.

### Determination of Lipid Droplet (LD) Contents

After three washes in 0.1% PVA-DPBS, DOs were fixed in 4% PFA for 30 min at RT and subsequently incubated in DPBS containing 10 μg/mL BODIPY 493/503 for 30 min at RT. After three washes with 0.1% PVA-DPBS, images were captured under a fluorescence microscope (Nikon, Japan) with the same scanning setting. The fluorescence intensity was calculated by ImageJ software.

### Annexin-V Staining

DOs were stained with an Annexin-V staining kit (Beyotime Biotechnology, Shanghai, China) according to the manufacturer’s instructions. After three washes in 0.1% PVA-DPBS, the oocytes were stained with 90 μL of binding buffer containing 10 μL of Annexin V-FITC for 30 min in a dark environment. After three washes with 0.1% PVA-DPBS, the oocytes were mounted on glass slides and captured under a confocal microscope with the same scanning setting.

### Evaluation of ROS Levels

The intracellular ROS levels in oocytes were detected with an ROS assay kit (Beyotime Biotechnology, Shanghai, China). DOs were incubated with 10 μM dichlorofluorescein diacetate (DCFH-DA; fluorescent probe) for 30 min at 38.5°C. After three washes with 0.1% BSA-DPBS, images were captured under a fluorescence microscope (Nikon, Japan). The fluorescence intensity was calculated with ImageJ software.

### RNA Isolation and Quantitative Real-Time PCR (qRT-PCR)

Pools of 100 oocytes/sample were collected, and then total RNA was isolated with an RNAprep Pure Micro Kit (Tiangen, China) according to the manufacturer’s instructions. cDNA was synthesized using a first-strand synthesis cDNA kit with random primers (Promega, United States). qRT-PCR was performed according to our published report (Nie et al., 2020). *GAPDH* was used as a reference gene, and the relative expression levels of target genes were determined by the 2^–ΔΔCt^ method. The primers used were as follows: *GAPDH*: F: 5′-AAGTTCCACGGCACAGTCAAG-3′, R: 5′-CACCAGCATCA CCCCATTT-3′; *CAT*: F: 5′-ACACACCTGAAGGATCCGGA-3′, R: 5′-AACCAGCTTGAAAGTGTGCG-3′; *GPx1*: F: 5′-GAAGTGTGAGGTGAATGGCG-3′, R: 5′-CTCGAAGT TCCATGCGATGT-3′.

### Statistical Analysis

The data are expressed as the means ± SEM. Prism 7 software (GraphPad, San Diego, CA, United States) was used to analyze the data via one-way ANOVA. Fluorescence intensity was calculated using NIH ImageJ software. Significance was accepted at *P* < 0.05.

## Results

### Protective Effects of MV on Meiotic Maturation and Subsequent Embryonic Development in LPS-Exposed Oocytes

**FIGURE 1:**
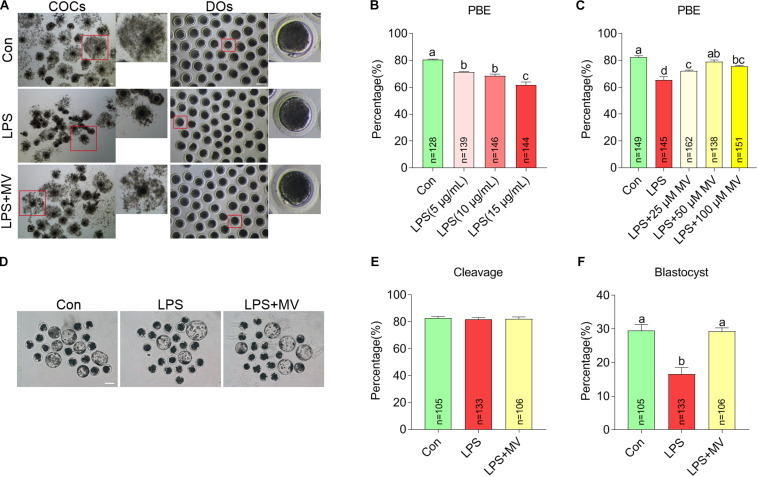
Effects of different concentrations of LPS and MV on meiotic progression and embryonic developmental potential in porcine oocytes. **(A)** Representative images of cumulus expansion and first polar body extrusion (PBE) in the control, LPS, and LPS + MV groups. **(B)** PBE rates in the control and LPS-exposed groups. **(C)** PBE rates in the control, LPS, and LPS + MV groups. **(D)** Representative images of blastocysts in the control, LPS, and LPS + MV groups. **(E)** Cleavage rates in the control, LPS, and LPS + MV groups. **(F)** Blastocyst formation rates in the control, LPS, and LPS + MV groups. Scale bar = 100 μm. COCs, Cumulus–oocyte complexes; DOs, denuded oocytes. “n” indicates the number of oocytes used. The data are presented as the mean ± SEM from at least three independent experiments. Different letters denote significant differences (*P* < 0.05).

To explore whether MV protects against the meiotic arrest caused by LPS, MV and LPS were added to the IVM medium. As shown in Figures 1A,B, the percentage of oocytes with first polar body extrusion (PBE) significantly decreased with increasing LPS concentrations (5, 10, and 15 μg/mL). Then, we supplied different concentrations of MV to IVM medium containing 15 μg/mL LPS. As shown in Figures 1A,C, the PBE ratio in LPS-exposed oocytes markedly increased with increasing MV concentrations (25, 50, and 100 μM). Because the PBE ratio in the 15 μg/mL LPS + 50 μM MV group were close to the control group, these concentrations were used in the subsequent experiments.

We next detected the effect of MV on embryonic development in LPS-exposed oocytes. As shown in Figures 1D–F, after PA, MV did not alter the cleavage rate but significantly increased blastocyst formation in LPS-exposed oocytes. Taken together, the results indicate that MV supplementation protects against oocyte meiotic maturation failure and embryonic developmental arrest caused by LPS exposure.

### MV Alleviates Abnormalities in Spindle Assembly, Chromosome Alignment, and α-Tubulin Acetylation in LPS-Exposed Oocytes

**FIGURE 2:**
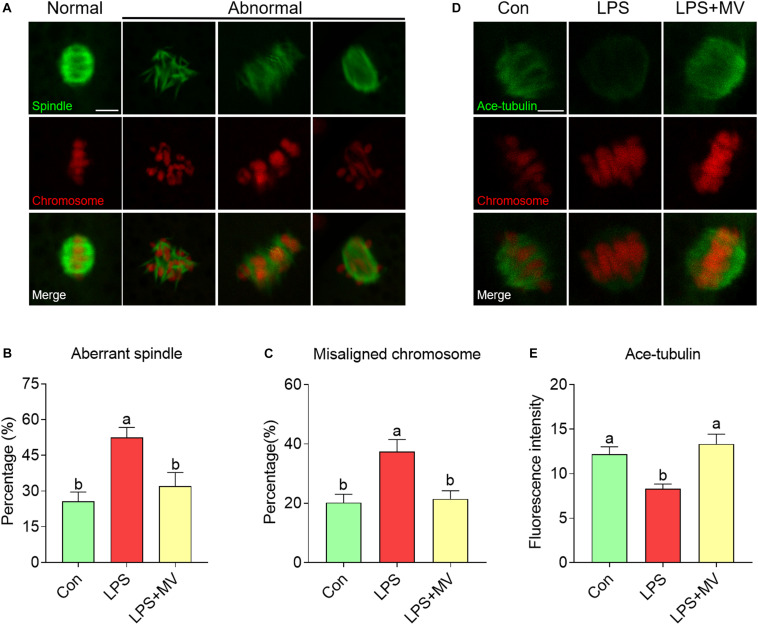
Effects of MV on spindle assembly, chromosome alignment, and α-tubulin acetylation in LPS-exposed porcine oocytes. **(A)** Representative images of normal/abnormal spindle (green) formation and chromosome (red) alignment in the oocytes. Scale bar = 10 μm. **(B)** Aberrant spindle rates in the control, LPS, and LPS + MV groups. **(C)** Misaligned chromosome rates in the control, LPS, and LPS + MV groups. **(D)** Representative images of acetylated α-tubulin (green) and chromosomes (red) in the control, LPS, and LPS + MV groups. Scale bar = 7.5 μm. **(E)** Quantitative analysis of the fluorescence intensity of acetylated α-tubulin in the control, LPS, and LPS + MV groups. The data are presented as the mean ± SEM from at least three independent experiments. Different letters denote significant differences (*P* < 0.05).

As shown in Figure 2A, under normal conditions, oocytes displayed barrel-shaped spindles with well-aligned chromosomes on the equatorial plate. A large proportion of aberrant spindles (Con: 25.71 ± 3.94%, *n* = 74 vs. LPS: 52.46 ± 4.27%, *n* = 86, *P* < 0.005) and misaligned chromosomes (Con: 20.19 ± 2.88%, *n* = 74 vs. LPS: 37.38 ± 4.10%, *n* = 86, *P* < 0.01; Figures 2B,C) were detected in LPS-exposed oocytes compared with control oocytes. However, MV significantly reduced the abnormal spindle rate (LPS: 52.46 ± 4.27%, *n* = 86 vs. LPS + MV: 32.05 ± 5.75%, *n* = 75, *P* < 0.05) and abnormal chromosome rate (LPS: 37.38 ± 4.10%, *n* = 86 vs. LPS + MV: 21.46 ± 2.77%, *n* = 75, *P* < 0.05; Figures 2B,C) in LPS-exposed oocytes.

Then, we detected the levels of α-tubulin acetylation, which determines the stability of α-tubulin. As shown in Figures 2D,E, the fluorescence intensity of acetylated tubulin was significantly lower in LPS-exposed oocytes than in control oocytes (Con: 12.17 ± 0.85, *n* = 40 vs. LPS: 8.29 ± 0.54, *n* = 43, *P* < 0.005). Interestingly, MV significantly increased the fluorescence intensity of acetylated tubulin in LPS-exposed oocytes (LPS: 8.29 ± 0.54, *n* = 43 vs. LPS + MV: 13.33 ± 1.10, *n* = 37, *P* < 0.0005). Taken together, the above results suggest that LPS impairs microtubule stability and spindle assembly in porcine oocytes, whereas MV supplementation ameliorates these impairments.

### MV Attenuates the Actin Polymerization Defects in LPS-Exposed Oocytes

**FIGURE 3:**
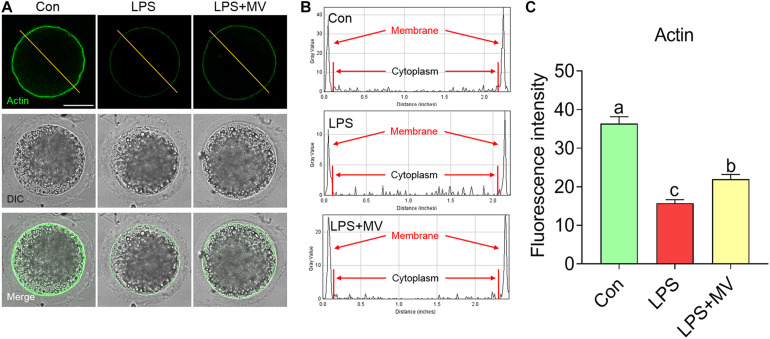
Effect of MV on actin polymerization in LPS-exposed porcine oocytes. **(A)** Representative images of actin (green) in the control, LPS, and LPS + MV groups. **(B)** Actin fluorescence distribution on the cell membrane and cytoplasm of oocytes in the control, LPS, and LPS + MV groups. **(C)** Quantitative analysis of the fluorescence intensity of actin in the control, LPS, and LPS + MV groups. Scale bar = 50 μm. The data are presented as the mean ± SEM from at least three independent experiments. Different letters denote significant differences (*P* < 0.05).

We next detected the actin distribution in both the membrane and cytoplasm of oocytes. As shown in Figures 3A–C, strong actin signals were observed on the plasma membrane in control oocytes, but the signals were weak in LPS-exposed oocytes (Con: 36.34 ± 1.82, *n* = 75 vs. LPS: 15.73 ± 0.93, *n* = 75, *P* < 0.0001). As expected, actin signals were significantly increased after MV supplementation in LPS-exposed oocytes (LPS: 15.73 ± 0.93, *n* = 75 vs. LPS + MV: 21.95 ± 1.23, *n* = 71, *P* < 0.005). Therefore, the above results indicate that MV supplementation ameliorates the actin polymerization defects induced by LPS.

### MV Alleviates the Change in Mitochondrial Contents and LD Contents in LPS-Exposed Oocytes

**FIGURE 4:**
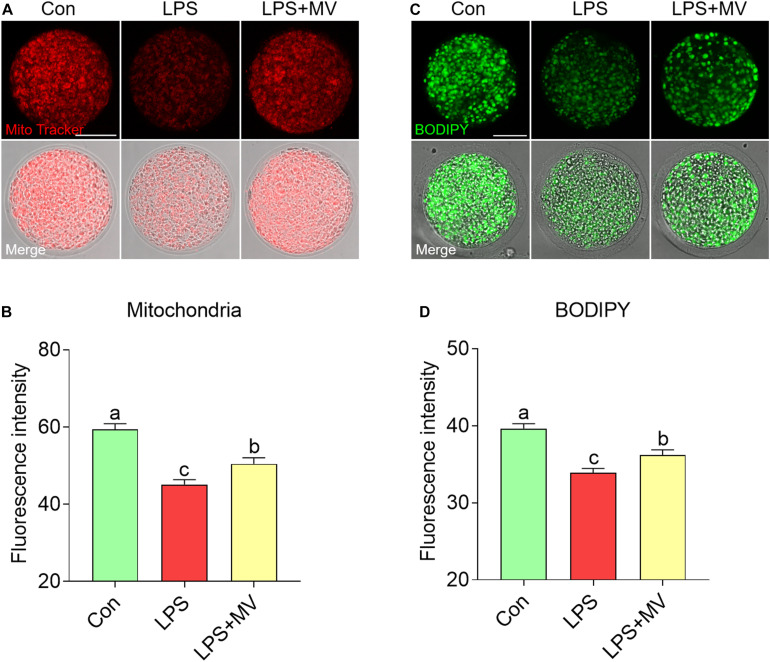
Effects of MV on mitochondrial content and LD numbers in LPS-exposed porcine oocytes. **(A)** Representative images of mitochondrial distribution in the control, LPS, and LPS + MV groups. Oocytes were stained with MitoTracker to visualize the mitochondria. **(B)** Quantitative analysis of the fluorescence intensity of MitoTracker in the control, LPS, and LPS + MV groups. **(C)** Representative images of lipid droplet (LD) distribution in the control, LPS, and LPS + MV groups. Oocytes were labeled with the lipophilic dye BODIPY^TM^ 493/503 to visualize the LDs. **(D)** Quantitative analysis of the fluorescence intensity of BODIPY in the control, LPS, and LPS + MV groups. Scale bar = 50 μm. The data are presented as the mean ± SEM from at least three independent experiments. Different letters denote significant differences (*P* < 0.05).

As shown in Figures 4A,B, compared with control oocytes, LPS-exposed oocytes exhibited significantly decreased mitochondrial contents, but MV supplementation significantly increased the mitochondrial contents (Con: 59.44 ± 1.42, *n* = 60 vs. LPS: 45.09 ± 1.27, *n* = 63, *P* < 0.0001; vs. LPS + MV: 50.47 ± 1.57, *n* = 60, *P* < 0.0001). Then, we examined the LD contents. As shown in Figures 4C,D, MV markedly increased the LD contents in LPS-exposed oocytes (Con: 39.65 ± 0.60, *n* = 67 vs. LPS: 33.89 ± 0.57, *n* = 67, *P* < 0.0001; vs. LPS + MV: 36.23 ± 0.65, *n* = 62, *P* < 0.0005). Collectively, these results indicate that MV attenuates the mitochondrial and lipid contents reduction caused by LPS exposure.

### MV Maintains m^6^A Levels in LPS-Exposed Oocytes

**FIGURE 5:**
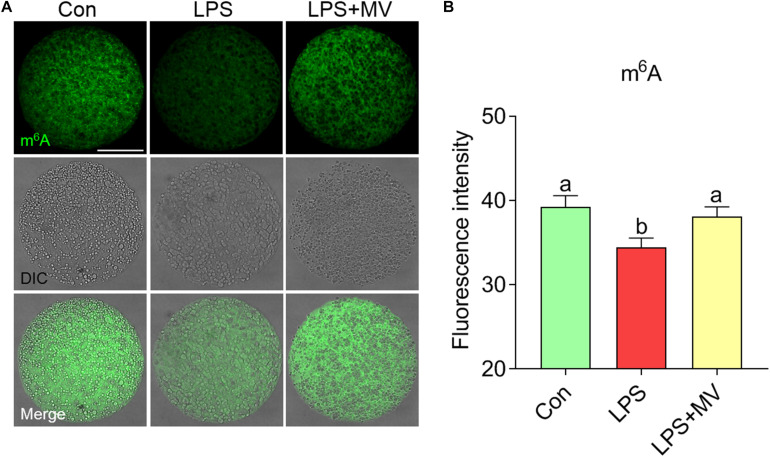
Effect of MV on m^6^A modification in LPS-exposed porcine oocytes. **(A)** Representative images of m^6^A signals (green) in the control, LPS, and LPS + MV groups. Oocytes were stained with an anti-m^6^A antibody. **(B)** Quantitative analysis of the fluorescence intensity of m^6^A in the control, LPS, and LPS + MV groups. Scale bar = 50 μm. The data are presented as the mean ± SEM from at least three independent experiments. Different letters denote significant differences (*P* < 0.05).

We next evaluated the effects of LPS and MV on m^6^A levels via immunofluorescence staining. As shown in Figures 5A,B, the fluorescence intensity of m^6^A was significantly reduced in LPS-exposed oocytes compared to control oocytes (Con: 39.26 ± 1.31, *n* = 80 vs. LPS: 34.41 ± 1.15, *n* = 83, *P* < 0.005). However, the fluorescence intensity of m^6^A was obviously increased after MV supplementation in LPS-exposed oocytes (LPS: 34.41 ± 1.15, *n* = 83 vs. LPS + MV: 38.09 ± 1.16, *n* = 81, *P* < 0.05). These results indicate that MV supplementation can maintain m^6^A levels in LPS-exposed oocytes.

### MV Reduces ROS Levels and Early Apoptosis in LPS-Exposed Oocytes

**FIGURE 6:**
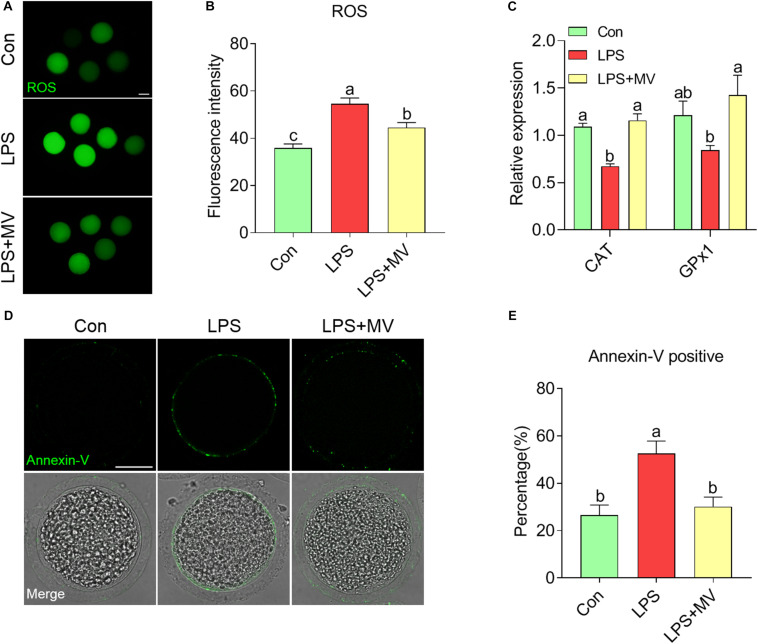
Effects of MV on ROS levels and apoptosis in LPS-exposed porcine oocytes. **(A)** Representative images of ROS content in the control, LPS, and LPS + MV groups. Oocytes were stained with DCFH-DA. **(B)** Quantitative analysis of the fluorescence intensity of DCFH-DA in the control, LPS, and LPS + MV groups. **(C)** Levels of two antioxidant enzyme genes (*CAT* and *GPx1*) in the control, LPS, and LPS + MV groups. **(D)** Representative images of apoptotic oocytes in the control, LPS, and LPS + MV groups. Oocytes were stained with Annexin-V-FITC. **(E)** The rate of early apoptosis in the control, LPS, and LPS + MV groups. Scale bar = 50 μm. The data are presented as the mean ± SEM from at least three independent experiments. Different letters denote significant differences (*P* < 0.05).

As shown in Figures 6A and B, ROS levels were obviously higher in LPS-exposed oocytes than in control oocytes (Con: 35.86 ± 1.77, *n* = 56 vs. LPS: 54.53 ± 2.55, *n* = 49, *P* < 0.0001), but MV effectively reduced ROS levels (LPS: 54.53 ± 2.55, *n* = 49 vs. LPS + MV: 44.51 ± 2.15, *n* = 52, *P* < 0.005). In addition, the relative expression of catalase (*CAT*) and glutathione peroxidase (*GPx1*) was significantly higher in LPS + MV oocytes than in LPS-exposed oocytes (*CAT* LPS + MV: 1.16 ± 0.072 vs. LPS: 0.67 ± 0.03, *P* < 0.0005; *GPx1* LPS + MV: 1.43 ± 0.20 vs. LPS: 0.84 ± 0.05, *P* < 0.0005; Figure 6C). As shown in Figures 6D,E, the percentage of Annexin-V-positive oocytes was obviously reduced after MV treatment in LPS-exposed oocytes (Con: 26.53 ± 4.30%, *n* = 83 vs. LPS: 52.61 ± 5.25%, *n* = 86, *P* < 0.005; vs. LPS + MV: 30.16 ± 4.00%, *n* = 89, *P* > 0.05). Collectively, these results demonstrate that MV inhibits the occurrence of oxidative stress and early apoptosis in LPS-exposed oocytes.

## Discussion

In this study, we found that MV significantly ameliorated porcine oocyte IVM inhibition due to LPS exposure. Our findings demonstrate that LPS exposure reduced meiotic maturation and embryonic development of porcine oocytes, associated with defective cytoskeletal dynamics, altered m^6^A levels, and decreased mitochondrial and LD contents. Moreover, LPS induced excessive ROS accumulation, consequently resulting in early apoptosis. Interestingly, MV alleviated the abovementioned defective parameters induced by LPS exposure.

Lipopolysaccharide (LPS) presents in follicular fluid of the cows with mastitis and endometritis (Magata et al., 2015; Roth et al., 2015; Heidari et al., 2019), indicating that it might directly inhibit oocyte maturation as the ovary is the niche of oogenesis. Actually, LPS exhibits negative effects on follicle development, oocyte maturation, and blastocyst formation in cows and mice (Shepel et al., 2018; Roth et al., 2020). Our results show that LPS suppressed porcine oocyte IVM and resulting blastocyst formation, consistent with the previous reports regarding cows and mice. Then, we applied the porcine oocyte as a study model and found that MV treatment evidently alleviated the LPS-induced meiotic defects. Similarly, we previously revealed that MV has protective and promotive effects on porcine oocyte maturation and competence (Nie et al., 2019, 2020). Thus, our findings suggest that MV is a candidate agent for amelioration of LPS-induced oocyte meiotic defects.

The cytoskeleton structure, which includes microtubules and actin filaments, is an indispensable component of meiotic maturation (Duan and Sun, 2019). Consistent with the findings of previous studies in bovine oocytes (Zhao et al., 2017), we found that LPS exposure induced porcine oocyte meiotic arrest by disrupting spindle assembly and chromosome alignment. Interestingly, MV supplementation protected against microtubule assembly and spindle defects in LPS-treated oocytes. Low acetylation levels at tubulin may destabilize microtubules and cause abnormal spindle assembly (Xu et al., 2017). Notably, we observed that MV restored α-tubulin acetylation to normal levels in LPS-exposed oocytes. Additionally, our results show that MV can rescue the compromised actin filament distribution in LPS-exposed oocytes. Therefore, this study suggests that MV supplementation protects against meiotic arrest and ameliorates cytoskeletal organization defects in LPS-exposed oocytes.

Mitochondria are essential organelles located in the cytoplasm, and mitochondrial integrity is regarded as a vital marker that can be used to evaluate oocyte cytoplasmic maturation (Babayev and Seli, 2015). Our results demonstrate that LPS caused reduced mitochondrial contents in porcine oocytes, consistent with a previous study on bovine oocytes (Magata and Shimizu, 2017). Likewise, we found that LPS dramatically decreased LD quantity in the oocytes. Mitochondria and LDs colocalize considerably in oocytes, and their interaction plays important roles in oocyte maturation and embryonic development (Holt et al., 1983; Dunning et al., 2010; Paczkowski et al., 2014). The neutral lipids liberated from LDs into the cytoplasm as free fatty acids are taken up into mitochondria for metabolism (Bradley and Swann, 2019). MV could preserve mitochondrial and LD contents in LPS-exposure oocytes; this was essential to oocyte meiotic maturation and competence.

We observed that oocyte m^6^A levels were decreased after LPS exposure and MV restored the changes. Currently, we do not know the exact reason why m^6^A levels were reduced by LPS exposure, but this defect could be attenuated by MV. It is possible that MV maintains RNA methyltransferase and eraser enzymes activity in the oocytes. In addition, oxidative stress also can induce the changes in epigenetic modifications (Guillaumet-Adkins et al., 2017; Tharmalingam et al., 2017). As expected, we observed that MV reduced ROS levels and increased the expression of antioxidant genes (*CAT* and *GPx1*) in LPS-exposed oocytes. Excessive ROS accumulation originating from the external microenvironment and cellular metabolism may cause oxidative stress and further induce early apoptosis in oocytes (Pandey et al., 2010; Jia et al., 2019; Zhang et al., 2020). Moreover, we found that MV reduced the occurrence of early apoptosis caused by LPS exposure. This result is similar to that of the previous studies showing that MV protects cells from oxidative stress (Li et al., 2014; Liu et al., 2018; Nie et al., 2019, 2020). Given our findings and those of the others, we speculate that MV reverses meiotic defects in LPS-exposed oocytes in part by preventing oxidative stress and early apoptosis.

In summary, our study demonstrates that LPS induces oxidative stress and early apoptosis and further disrupts cytoskeleton dynamics, organelle integrity, and epigenetic modifications in porcine oocytes. However, MV administration can reverse the adverse parameters resulting from LPS exposure. Notedly, MV can improve IVM and embryonic developmental potential of LPS-exposed oocytes. The underlying mechanisms of the proactive role of MV in LPS-exposed oocytes still need to be further studied.

## Data Availability Statement

All datasets generated for this study are included in the article/supplementary material, further inquiries can be directed to the corresponding author/s.

## Ethics Statement

The animal experiments were approved by the Institutional Animal Care and Use Committee (IACUC) of Guangxi University and were conducted in accordance with the animal welfare and ethical rules.

## Author Contributions

KY and XL conceived the study. KY, KC, JN, LS, HYZ, and HTZ performed the experiments. KY, C-LX, XY, and XL analyzed the data and wrote the manuscript. All the authors reviewed the manuscript, approved the final version of the manuscript, contributed to the article, and approved the submitted version.

## Conflict of Interest

The authors declare that the research was conducted in the absence of any commercial or financial relationships that could be construed as a potential conflict of interest.
